# Anæsthesia—Who First Demonstrated Its Practicability? And Who Was the First to Bring It into Use

**Published:** 1864-02

**Authors:** 


### Selections.

ANÆSTHESIA—WHO FIRST DEMONSTRATED ITS PRACTICABILITY? AND WHO WAS THE FIRST TO BRING IT INTO USE—*By a Lover of Truth.*—It is well known that Wm. T. G. Morton has been for several years harassing the two Houses of Congress with importunities for a national recognition and reward for having conferred on humanity by far the greatest boon which has originated with the present century. In short, he claims to be entitled to the sole credit of being the first to discover the practicability of rendering the human system insensible to pain under surgical operations, and the first to apply with success, adequate means to that end. The state or condition to which the system is reduced, is called anæsthesia and the meaus used to produce that state anæsthetic agents. Those most employed are, nitrous oxyd gas, sulphuric ether, chloroform, and chloric ether, and the process is inhalation.

It is difficult to overrate the value or importance of this discovery, and that our country is entitled to the honor of having made it, is admitted by the whole civilized world; but when we come to descend from a whole people to the individual author, the question arises whether such author is to be found in Wm. T. G. Morton. Is it to him that we are indebted for detecting and revealing this great secret of nature? If so, then no doubt he should be regarded as a public benefactor, and should be abundantly honored and rewarded as such. He should be taken from comparitive obscurity and raised to a position not less exalted than that occupied by the immortal JENNER, and the national treasury should be open to his requisitions to such an extent as to place him far beyond want, if not in affluence. But did anæsthesia, in the modern sense of that word, originate with Wm. T. G. Morton? Was the great fact detected by his vigilance, or developed by his experimentation, or is he an impostor who has bedecked himself with the plumes of another, and whose claims should be rejected with contempt if not with indignation?

On the 10th day of December, 1844, there resided in the City of Hartford, Conn, a citizen named Horace Wells, a native of New Hampshire, though he had made Hartford his home for a considerable number of years, a surgeon dentist by profession, who possesed a quick eye and an acute mind with a philosophic turn, who was ardent, enthusiastic, susceptible, genial, and in every respect trustworthy, and whose physical constitution was as delicate as his moral and intellectual nature was sensitive. No man ever enjoyed the confidence of a community more entirely than he did that of Hartford. Enmity did not know him, and friendship and esteem every where attended his foosteps.

On the evening of this same 10th of December, 1844, this worthy man with his lady, attended at Hartford, a chemical lecture by Dr. G. Q. Colton, during or after which he (Dr .C.) administered to Dr. Wells, Samuel A. Cooley, and several other persons, the nitrous oxyd gas. Mr. Cooley on being brought under its influence, became unusually excited, and taking the floor performed sundry evolutions and gyrations thereon, during which he contused and abraded both of his knees pretty extensively by collisions with the benches, which fact was noticed by Dr. Wells. On recovering his self-possession the Doctor inquired of him whether he felt any pain from the injuries he had received. He replied he was not conscious of having sustained any injury, but on pulling up his pantaloons blood appeared in profusion. Wells immediately turned to a friend sitting by, and expressed a belief that a man could by inhaling the gas, render hinself so insensible that he could have a tooth extracted without pain. While escorting his wife home he reiterated an expression of the same belief and again reiterated it to a brother dentist and a particular friend, on whom he called the same evening to canvas the subject, Having spent some time in considering the matter, Dr. Wells declared it to be his purpose to take the gas the next day and have a defective tooth (a large molar) extracted, and thus to test the correctness of his theory.

That is right exclaimed his friend. *It is but just that we should commence with experimenting on ourselves.* Accordingly, the next morning Wells called on Dr. Colton, stated the facts which had arrested his attention, and his conclusions therefrom. He requested him (C.) to take a bag of the gas over to the office of the dentist already alluded to, which he did accordingly. On the parties there assembling Wells put himself in the operating chair. Colton administered the gas, and as soon as he was brought under its influence the dentist extracted the tooth, and Wells on recovering his consciousness, exclaimed "*A new era in tooth-pulling! It did not hurt me more than the prick of a pin!*"

There are not a few persons of high intelligence who have given this subject a particular consideration, who hold that there was here not only a conception of the anæsthetic idea but an actual parturition of it. That this offspring of genius was then and there brought into the world perfect and complete in all its proportions and parts, and that the strenous endeavors which have since been made elsewhere to foist on the public other heirs to this inheritance of fame, can but be regarded as a base attempt to substitnte for the true and the legitimate, the spurious and the illegitimate. I, however, purposely withhold my opinions for the present.

On the 30th of September, 1846, there resided and still resides in Boston, Mass., the same Wm. T. G. Morton already named, also a surgeon-dentist, whose characteristics I do not propose to describe here, as your readers will be much better qualified to appreciate the justness and propriety of my strictures after they shall have been put in possession of facts which will subsequently appear. "Toward evening of the day last named (we use the language of Morton) a man residing in Boston, came in (meaning his office) suffering great pain and wishing to have a tooth extracted, and asked if he could be mesmerized." He (M.) told him that he "had something better, and saturating his handkerchief (meaning with sulphuric ether) he "gave it to him to inhale. He became unconscious almost immediately. It was dark, and Dr. Hayden held the lamp while" he "extracted a firmly rooted bicuspid tooth. There was not much alteration in the pulse and no relaxation of the muscles. He recovered in a minute and knew nothing of what had been done to him. He remained for some time talking about the experiment." This he (M.) considered "to be the first demonstration of this new feat in science." Such was the language used by Morton himself, in his memoir to the Academy of Arts and Sciences at Paris, describing his first anæsthetic experiment on a human being, (changing only the first person singular into the third) and it is but candid to admit that I fully believe that the agent used was adequate and produced the effect desired, and that the man's tooth (whose name was Eben Frost) was extracted without pain, and therefore that he (M.) succeeded in performing on the occasion named, a genuine anæsthetic operation, but your readers need be in no haste to conclude with Morton that it was "*the first* demonstration of this new feat in science."

On the same 30th day of September, 1846, there resided and still resides in Boston another citizen named Charles T. Jackson, a distinguished physician and surgeon and a learned professor, whose name, position, and character for high ability and many accomplishments are familiar to the intelligent public in the United States and not unknown to the savans of Europe. Like, however, many other men of mark, he is not without his idiosyncracies—his bump of self-esteem would seem to be pretty extensively developed, and, perhaps, your readers may, in view of facts hereafter to appear, be disposed to inquire whether a morbid love of fame has not betrayed him into a line of conduct not very creditable to his good sense, to say nothing of his fairness, candor, and rectitude. For myself I am free to admit that I am disposed to exercise in this case much of that charity which is said to "cover a multitude of sins."

In the winter of 1842, he (J.) had prepared, as he says, a large quantity of chlorine gas, and one of the jars containing it was overturned and broken. In trying to save the vessel he accidentally inhaled and filled his lungs with the gas which nearly suffocated him and endangered his life. His throat and lungs being greatly inflamed, he concluded the next morning to seek relief by inhaling the vapor of ether, and this he accordingly did. He realized his object and the effect of the vapor was to throw him into a state of insensibility. "Reflecting on the phenomena the idea flashed on his mind, (I am using the words of Dr. J.) that he had made the discovery which he had so long been in quest of, the means of rendering the nerves of sensation so insensible as to admit of the performance of a surgical operation without pain." The learned Dr. insists that there was on this occasion a distinct conception of the anæsthetic idea, but he admits that it lay inert in the womb of mind, (if I may be allowed such an expression) and that there were no travail throes for years, but at length Dr. Morton, on this same 30th of September, called on him making inquiries touching the nitrous oxyd as an agent for producing insensibility when (as J. claims) he entered at once upon the subject of anæsthesia, told Morton to substitute the vapor of sulphuric ether for the nitrous oxyd and then gave him full information as to its nature and effects, and precise instructions as to the method of using it. In short, Dr. Jackson strenuously insists that in a scientific point of view, Morton in operating in the Frost case acted merely as his substitute or agent, and that the result should in justice, be passed to his credit. He affirms that Morton was profoundly ignorant and utterly incapable of scientific investigation. But how he came to commit the development of an idea which according to his statements he had so long entertained, and which was of such vast consequence to humanity to such a character, he has not as yet explained. If Dr. J. will now so far modify his pretensions as to say that Morton got whatever he knew of the practicability of anæsthesia from Wells, and that he (J.) on being informed by the former of the use which the latter was making of the nitrous oxyd, at once told him to substitute therefor the vapor of sulphuric ether, and gave him full information as to its nature and effect, and instructions as to the method of using it, I believe that he will place himself on ground both truthful and respectable, which he should have occupied from the beginning. I declare it to be my unqualified belief that Morton did nothing and was not capable of doing anything in the Frost case except *that* and precisely *that* which Dr. Jackson told him to do. Whether such fact will authorize the latter to arrogate to himself the name and character of discoverer of anæsthesia and author of this great movement, will be considered hereafter.

I now come to a series of transactions of a most significant character which have not as yet been distinctly presented to the public, certainly not consecutively, nor has their bearing on the main question been shown.

As on one occasion "an idea flashed on the mind" of Dr. Jackson, so on a different occasion another idea flashed on the mind of the illustrious Morton. No sooner had the tooth of the happy Frost been extracted (we call him happy on account of the exquisite enjoyment which ever attends the anæsthetic state) than the aforesaid "flash" prompted Morton to the conclusion that his pretended discovery might avail "to put money in his purse." The very next day he, with that object in view, hied to the office of R. H. Eddy, Esq., a very respectable patent lawyer residing in Boston, to whom he stated his case and asked his professional aid in obtaining letters patent of the United States for his supposed discovery. Mr. Eddy proceeded to inquire minutely into the matter and soon ascertained that Dr. Jackson was most intimately connected with the subject. The Dr. as he concluded had furnished mind, intelligence, and science, and Morton, sinews, muscles and thews, and the result of these conjoined powers was the ejection of Frost's tooth without a particle of pain.

Can this be said (meditated Eddy) to have resulted from the part taken by either Jackson or Morton alone? If the latter were to take the patent out, would it not be proved conclusively that he acted implicitly in conformity with the instructions of the former; and if, on the other hand, Jackson were to take it out, would it not be proved with equal clearness, that Morton alone performed the experiment, and that Jackson was not even present. Mr. Eddy concluded to take time for consideration and did not act finally on the subject till near the close of the month. In the meanwhile he saw Dr. Jackson and had from him a full confirmation of what Morton had conceded, to-wit: that he (J.) had participated in the affair in the manner stated, and was entitled to share in the honors and rewards of the discovery if any discovery there was.

On the 27th of October the parties met at the office of Eddy, and he then announced to them a decided conviction that both must join in the application for the patent to make it valid. In the first instance Jackson objected from an apprehension that he might thereby expose himself to the censure of the Massachusetts Medical Society, and in consequence might even be expelled, but Mr. Eddy succeeded at length in overcoming his scruples. The parties being at full accord, Mr. Eddy proceeded to draw up the papers, which were: 1. An assignment by Jackson to Morton of all his "right, title, and interest" in what he designates as "a new and useful improvement in surgery," and 2. An application for a patent with specifications thereto attached in the usual form to be signed and sworn to by both.

The assignment commences with a preamble reciting as follows: "Whereas I, Charles T. Jackson, of Boston, in the State of Massachusetts, chemist, have in conjunction with Wm. T. G. Morton of said city, dentist, invented or discovered a new and useful improvement in surgical operations on animals, whereby we are enabled to accomplish many if not all operations on animals such as are usually attended with more or less pain and suffering, without any or very little pain or muscular action to persons who undergo the same, and whereas the said Morton is desirous of procuring a patent for the same, and whereas *I am desirous of benefiting him and not to be interested in any patent*, I have, therefore, in consideration of one dollar," etc. The instrument then proceeds in the usual form to "assign, set over, and convey" to Morton "all the right, title, and interest" of him (J.) "in the said invention and discovery, etc.," declaring that he had that day signed and executed the specifications "*in conjunction* with" Morton, "for the purpose of enabling him to obtain a patent thereon" and requesting the Commissioner to issue the same to Morton "in *his name* and as *his* (Jackson's) *assignee.*" This paper bears date October 27th, 1846.

The application for the patent was signed by both and bears the same date. The specifications annexed were made in the names of both and throughout the whole document they speak of the supposed improvement or discovery as the fruit of their united endeavors and joint efforts. It begins thus: "be it known that we Charles T. Jackson and Wm. T. G. Morton, of Boston," etc., "have invented or discovered a new and useful improvement in surgical operations whereby *we* are enabled," etc. They speak of "*our* discovery"—"this is *our* discovery." "Constitutes *our* invention"—"from the experiments *we* have made *we* are led to prefer," etc. Operating through the stomach they add "*we* consider in no respect to embody *our* invention as *we* operate through the lungs and air passages," etc., and then they conclude as follows: "what *we* claim as *our* invention is the hereinbefore described means by which *we* are enabled to effect the highly important improvement in surgical operations, viz.: by combining therewith the application of ether or the vapor thereof substantially as above specified."

Mr. Eddy having drawn up the papers, Professor Jackson signed and delivered the assignment, and both Jackson and Morton signed the specifications, and not only so, they both swore to it. The certificate of the Justice of the Peace who administered the oath, is as follows:

"STATE OF MASSACHUSETTS, } ss.

COUNTY OF SUFFOLK. }

"On this 27th day of October, A. D. 1846, personally appeared before me, Charles T. Jackson and Wm. T. G. Morton, *and made oath that they do verily believe themselves to be the original and first inventors of the improvement* hereinbefore discovered, that they do not know or believe the same to have ever before been known or used, and they are citizens of the United States of America. "R. H. EDDY,

*Justice of the Peace.*"

Morton thereupon transmitted the assignment and application for a patent with the specifications thereto annexed, and the certificate thereon indorsed to the Patent Office at Washington, and in due season letters patent were issued for a joint discovery and improvement to him partly in his own right and partly as the assignee of Jackson. These letters bear date on the 12th day of November, 1846. I have obtained copies of the papers duly authenticated, so that I know whereof I speak.

It should be stated here that in the report drawn up by the late Col. Bissell, Chairman of a Select Committee, House of Representatives, Second Session, 30th Congress, but not presented, though subsequently taken and printed by Morton, the above named assignment appears in a mutilated form. The whole preamble is left out and nothing inserted but the granting or assigning part of the instrument Who mutilated this document? It certainly could not have been Col. Bissell, as he was a gentleman of honor and quite above such a trick. It is obvious that Morton was deeply interested in excluding from Congress all knowledge of the "conjunction" so explicitly avowed in the preamble. It is the last thing he would be willing to have disclosed at Washington when in pursuit of a great national reward. This affair would seem to have a very squally look. Possibly Dr. Morton, dentist, can make a satisfactory explanatian, but I doubt it. (Vide the mutilated document as quoted in the report of the Hon. Mr. Wilson, from the Senate Committee on Military Affairs at the last session, p. 158.)

I further add that the patent expired on the 12th day of November, 1860. Morton applied for a renewal, Jackson refused to concur, and for this reason the Commissioner denied the application. Thus the "conjunction" of the two in making the discovery has at all times been recognized at the Patent Office.

Whether the supposed discovery or improvement was patentable, and whether Dr. Wells was not the first to detect the principle, and the first to bring an adequate process of development into use, are questions which I shall examine hereafter. In either case the patent was utterly null and void.

But the above papers do not disclose all that I have of a union of the efforts of Jackson and Morton to produce the result named. On the 28th day of November, 1846, Morton entered into copartnership with N. C. Keep, to practice dentistry at Boston. On the next day they caused an advertisement to be drawn up which Morton after a particular examination approved. It was signed by both, and on the same day (Nov. 29th) was published in three evening papers, one of which was the *Evening Traveler*. The advertisement is as follows:

"The subscribers having associated themselves in the business of dental surgery, would respectfully invite their friends to call on them at their rooms, No. 19 Tremont Row. They confidently believe that the increased facilities which their united experience will afford them of performing operations with elegance and dispatch, and the additional advantage of having them performed without pain by the use of *the fluid recently invented by Doctors* Jackson and Morton, will not only meet the wishes of their former patients, but secure to them additional patronage."

Ah! Dr. Morton! more "conjunction!" but what of *that fluid* which you and Jackson invented? Did your united powers *invent* Sulphuric Ether? Recollect, that *false pretences* in business transactions are *criminal!* in scientific, *detestable!* but here we have a "conjunction" of both!

According to the preamble of the assignment it would appear that Dr. Jackson was in this affair enacting a truly disinterested part—he was not willing "to be interested in any patent;" and not only so, but a highly beneficent part—he was "desirous of benefitting" Morton. But "all is not gold that glitters;" his disinterestedness and beneficence were nothing but outside show, for Jackson took from Morton at the same time for his "conjunction" in making this discovery, and for the assignment, his bond, obligating him to pay over to Jackson ten per cent. of the proceeds of the patent for his interest in it, and subsequently by his counsel he demanded twenty-five per cent. of the profits both at home and abroad, which Morton refused to concede.

I now come to a chapter as to the proceedings of Jackson, which I regret my obligations to the cause of justice and truth should constrain me to open. A few days after the supposed discovery, he (J.) drew up a formal paper setting forth the nature and particulars thereof, and claiming it to have been exclusively his own, and this paper sealed up, he forthwith forwarded to a friend in Paris, and directed him to lodge it in the archives of the Academy of Arts and Sciences there, unopened, till he should give further direction on the subject. This paper was as follows:

Boston, November 13th, 1846.

"I request permission to communicate through your medium to the Academy of Sciences a discovery which *I* have made, and which I believe important for the relief of suffering humanity as well as of great value to the surgical profession. Five or six years ago I noticed the peculiar state of insensibility into which the nervous system is thrown by the inhalation of the vapor of pure Sulphuric Ether, which I respired abundantly, first by way of experiment, and afterwards when I had a severe catarrh, caused by the inhalation of chlorine gas. I have latterly made a useful application of this fact by persuading a dentist of this city to administer the vapor of Ether to his patients when about to undergo the operation of extraction of teeth. It was observed that persons suffered no pain in the operation, and that no inconvenience resulted from the administration of the vapor."

Subsequently the Dr. addressed to his friend another letter, directing his first communication to be opened and its contents communicated to the Academy. The following is an extract from it:

December 1st, 1846.

"The advantage of the application of the vapor of Ether has been completely established in this country, and the agent has been used with great success in the Massachusetts General Hospital."

Ah! good Doctor! what has become of your "conjunction" with Morton in making this discovery? And how came it about after you had not only stated, stipulated, and even sworn, that this great secret of nature was detected by the joint efforts of Morton and yourself, and after you had conceded to him much the greater part of the proceeds, and had taken his bond for the balance, that you were making secret communications to a scientific body in Europe, appropriating to yourself the whole credit of bringing this deeply interesting fact to light?

It must be quite apparent to your readers that on the return news from Europe, as to the proceedings of Jackson at Paris, there could no longer be peace between the "high contracting powers." They immediately declared war against each other, and have prosecuted hostilities with intense bitterness from that day to this. If the result shall be (as in the case of the Kilkenny cats,) a complete annihilation of their respective claims, every lover of truth, honor and rectitude, must rejoice.

In conclusion (for the present), I will only ask your readers to consider in view of the revelations of these columns, how much of brass must enter into the composition of Wm. T. G. Morton's face to enable him to appear before Congress and demand a great national reward, on the ground that *he* is the sole discoverer of practical Anæsthesia, and, that to him, and to him only, is the world indebted for this priceless boon to humanity? "Conjunction!" "Conjunction!" should in conspicuous characters be burnt into his forehead, and impudence should in his case, if ever, be "chastised with scorpions!"

I shall treat of other branches of this subject hereafter.
—*Medical & Surg. Reporter.*

---

To Improve the Solubility of India Rubber.—Caoutchouc (virgin gum) becomes more soluble in oil of turpentine or benzine, if it has previously been macerated for some time with ammonia, until perfectly decolorized. When it is then washed with water, and dried, it will dissolve more readily in the menstrua named.—*Am. Drug. Circ.*

